# Advanced Atrio-Ventricular Blocks in a Foal Undergoing Surgical Bladder Repair: First Step to Cardiac Arrest?

**DOI:** 10.3389/fvets.2018.00096

**Published:** 2018-06-08

**Authors:** Vincent Marolf, Alessandro Mirra, Nathalie Fouché, Cristobal Navas de Solis

**Affiliations:** ^1^Department of Clinical Veterinary Science, Division of Anaesthesiology and Pain Treatment, Vetsuisse Faculty, University of Bern, Bern, Switzerland; ^2^Swiss Institute of Equine Medicine (ISME), Vetsuisse-Faculty, University of Bern and Agroscope, Bern, Switzerland

**Keywords:** uroperitoneum, bradycardia, atropine, arrhythmia, hypotension

## Abstract

A 3-day-old Swiss Warmblood colt was diagnosed with uroabdomen after urinary bladder rupture. The foal had classical electrolyte abnormalities (hyponatremia, hypochloremia and hyperkalemia) on presentation. The foal was supported prior to surgery with intravenous fluids and the electrolyte abnormalities were treated with physiologic saline, glucose and insulin. Urine could not be drained from the abdomen prior to surgery because the omentum was continuously occluding the drainage cannula and due to progressive abdominal distension, it was decided to pursue surgery without further correction of electrolyte abnormalities. After induction of anaesthesia, signs of hypoxemia were present. Controlled mandatory ventilation using a pressure-controlled ventilation mode with positive end-expiratory pressure was initiated. Urine was drained from the abdomen by free flow. Atrio-ventricular (AV) blocks unresponsive to intravenous antimuscarinic drugs developed. After low dose of epinephrine and cardiac massage, sinus rhythm was restored. Surgery was completed successfully and the foal recovered from anaesthesia. The postoperative period was uneventful and the foal was discharged from the hospital three days later. Based on a clinical case, the purpose of the manuscript is to provide the clinician with potential causes, prevention and treatment of this already known but rarely observed dysrhythmia which could lead to fatal consequences. Definitions of cardiac arrest and asystole are reappraised. We discuss the fact that advanced AV-blocks should be treated as a cardiovascular emergency with advanced life support. The early recognition of advanced AV blocks is the first step to reduce perioperative mortality and morbidity of foal suffering from uroabdomen.

## Description

**Figure 1:**
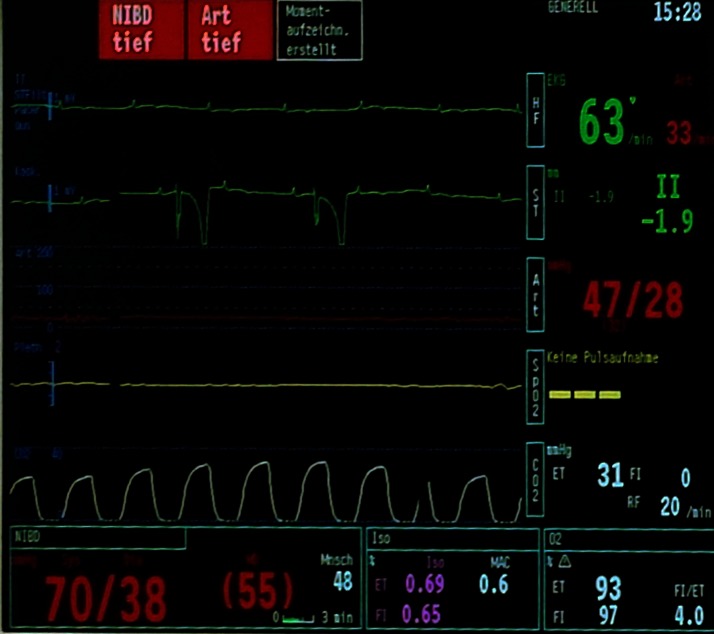
Monitor screen capture taken during anaesthesia of a foal undergoing surgical bladder repair. Advanced 2nd or 3rd degree atrioventricular blocks with intermittent ventricular contractions were observed. The pulse oximetry and invasive blood pressure measurement correlate with the top line of the electrocardiogram (ECG) and illustrate absent pulse pressure waves when ventricular rhythm was not recorded. The bottom line of the ECG was recorded before the top line.

A 3-day-old 60 kg Swiss Warmblood colt was presented to the university veterinary hospital approximately two hours after the onset of clinical symptoms observed by the owner in the morning. The foal was recumbent, tachycardic (160 beats/minute), tachypneic (60 breaths/minute), with abdominal distention, pale-pink mucous membranes and a capillary refill time of 2.5 s. Uroperitoneum due to a ruptured bladder was diagnosed with abdominal ultrasonography and peritoneal fluid aspirate analysis (yellow, cloudy aspirate; creatinine 1evel: 1,264 µmol/l). Surgical bladder repair after medical stabilization was planned. Complete blood count analyzed at initial presentation revealed leukocytosis with left shift, lymphopenia and monocytosis. Complete serum biochemistry revealed hyponatremia, hyperkalemia, hypochloremia, normocalcemia, (Na+: 107 mmol/l, K+: 4.9 mmol/l; Cl- 71 mmol/l, Ca2+: 3.24 mmol/l), hyperglycemia (11.71 mmol/l), azotemia (BUN: 10.01 mmol/l, creatinine: 327 µmol/l) and increased activities of liver enzymes. Packed cell volume and total solids were measured at 46% and 71.2 g/l; respectively. Fluid therapy with 3 litres of 0.9% NaCl given as bolus followed by 5% glucose solution in saline solution at 5 ml/kg/hr and an intramuscular injection of insulin 0.1 IU/kg (Novo Rapid, Novo Nordisk, Switzerland) were initiated. Abdominal distension increased due to inability to drain the uroabdomen as the omentum repeatedly occluded the abdominal drain. Plasma electrolytes, glucose and lactate were controlled after four hours (Na+: 113 mmol/l, K+: 5.9 mmol/l; Cl- 77 mmol/l, glucose: 9.4 mmol/l, lactate: 2.1 mmol/l) and it was decided to perform the surgery despite the electrolyte imbalances. The foal was premedicated with butorphanol 0.05 mg/kg IV (Morphasol, Dr. E. Graeub, Switzerland) and diazepam 0.1 mg/kg IV (Valium, Roche, Switzerland) and general anaesthesia was induced with ketamine 1 mg/kg IV (Narketan, Vetoquinol, Switzerland) and propofol 1.5 mg/kg IV (Propofol, Fresenius Kabi, Germany). The trachea was intubated with a 12 mm cuffed silicone endotracheal tube which was connected to a circle system anaesthesia machine (Fabius, Dräger Medical, Germany). Anaesthesia monitoring (multiparameter analyzer, Datex Ohmeda S5, GE Healthcare, USA) consisted of a 3-lead base apex ECG lead II derivation, hemoglobin saturation by pulse oximetry, end-tidal carbon dioxide (ETCO_2_) and inspired fraction of oxygen (FiO_2_) with gas analyzer and invasive and non-invasive blood pressure. Blood pressure was monitored with an inflatable cuff placed at the base of the front limb and with an invasive arterial 22-gauge catheter placed in the facial artery. Fluid therapy administered during anaesthesia was based of saline solution 0.9% (10 ml/kg/hr) supplemented with glucose 5% (glucose: 0.5 g/kg/hr). Anaesthesia was maintained with isoflurane (Attane Isoflurane, Provet, Switzerland) in oxygen with a fresh gas flow set a 2 L/min. The foal was breathing spontaneously and the first recorded pulse oximetry measurement indicated an oxygen saturation of 78% (FiO_2_ 0.87). Thus, five minutes after connection to the anaesthesia machine, controlled mandatory ventilation with high peak inspiratory pressure (PIP: 40 cmH_2_O) and positive end-expiratory pressure (PEEP: 5 cmH_2_O) was initiated. An arterial blood gas analysis was performed revealing following values: pH: 7.27; PaO_2_: 3.2 kPa, 61.9 mmHg; PaCO_2_: 6 kPa, 45.2 mmHg; HCO3-: 19.7 mmol/l; TCO2: 21.1 mmol/l; BE: −5.8; Lactate: 2.6 mmol/l; Na+: 113 mmol/l; K+: 4.9 mmol/l; Cl-: 81: mmol/l. One puff of nebulized salbutamol (100 mcg; Ventolin, GlaxoSmithKline AG, Switzerland) was delivered via endotracheal tube. Because of mild hypotension (invasive mean arterial pressure of 60 mmHg) dobutamine (Dobutrex, Teva Pharma, Switzerland) at 0.6 mcg/kg/min has been initiated. A normal sinus rhythm of 90 beats/minute was recorded before surgical incision. A midline abdominal incision was performed and approximately 10–12 liters of intraabdominal fluids were released by free flow over approximately thirty seconds. Cardiac dysrhythmia with long periods of absent atrioventricular conduction (see Figure 1) developed. P-P and P-R intervals did appear to be slightly irregular but each QRS complex was always preceded by a P-wave. QRS complexes did not appear wide and bizarre. Progressive decrease of the ETCO_2_ (from 6 kPa; 45 mmHg to 2.9 kPa; 22 mmHg) values were observed. Periods of advanced atrioventricular blocks were characterized by the absence of pulse oximeter and invasive blood pressure waves. After development of AV blocks, dobutamine administration was stopped and atropine 0.02 mg/kg IV (Atropinsulfat, Amino, Switzerland) was given. Because no improvements in the rhythm was observed, four additional doses of atropine were injected (0.01–0.02 mg/kg IV), but none was successful. Thereafter, a prolonged period of ventricular asystole occurred (approximately 30 s), intravenous epinephrine 0.01 mg/kg was administered, the isoflurane vaporizer was turned off, the foal placed in lateral recumbency and chest compressions (approximately 120/minute) were initiated. A sinus tachycardia at 130 beats/minute was observed. Normocapnia was restored within 30 s of cardiopulmonary resuscitation (CPR). Electrolytes concentrations (Na+: 116 mmol/l, K+: 6.3 mmol/l; Cl- 89 mmol/l) and arterial blood gas analysis (PaO2: 29.6 kPa, 222 mmHg; PaCO2: 7.8 kPa, 58.7 mmHg) were measured shortly after CPR. No more cardiac dysrhythmias were observed, surgery was completed within 100 min and total anaesthesia time was 160 min. Efforts to keep normothermia during anaesthesia included: use of a medical forced-air warming device, abdominal flushing with fluids at body temperature and administration of warm IV fluid therapy during anaesthesia. The lowest temperature was measured at the end of anaesthesia (37.4°C). The foal exhibited signs of excitement (paddling, shaking) during recovery which resolved after 10 min of manual restraint. The day after surgery, blood work was repeated, showing the following values (Na +128 mmol/l, K + 3.5 mmol/l, Cl- 109 mmol/l, iCa ++ 1.54 mmol/l, glucose 9.0 mmol/l, lactate 1.9 mmol/l). The postoperative period was uneventful and the foal was discharged three days later. Eighteen months later the owner reported that the foal had been healthy since discharge. Written signed owner consent had been obtained for publication of the present case.

## Discussion

Atrioventricular blocks are classified in first, second or third degree. First degree AV block is defined as a prolongation of PR interval (>500 ms in the adult horse). This implies that all atrial electrical signals are conducted to the ventricle despite being delayed. Second degree AV block is characterised by the intermittent absence of electrical conduction from the atria to the ventricles which result in P waves not followed by QRS complexes. This rhythm can be physiological and is often recorded in horses at rest. This type of block is further classified in Mobitz type I (Wenkebach phenomenon) or Mobitz type II. Type I is characterised by a progressive lengthening of the PR-Interval which is succinctly followed by a single isolated P wave. Type II is independent of the PR Interval which remains identical for all the complexes. Second degree AV block can also have severe hemodynamic consequences if succint P-waves are not conducted. It is then called “advanced” or “high-grade” 2nd degree AV block. Third degree AV block is defined by the absence of atrioventricular conduction. Atrial and ventricular electrical activities are dissociated and a ventricular escape rhythm often develops (1, 2).

The choice of discontinuing the continuous rate infusion of dobutamine was based on its possible arrhythmogenic properties (3). However, if the anaesthetist wishes to increase cardiac conduction and contractility, the right decision would be to increase the dose of administration. After careful consideration, the authors do believe that increasing the dose of dobutamine might remain a valid choice. Dopamine does also have arrhythmogenic properties and the use of a dose of ≥5 µg/kg/min has been suggested to treat advanced 2nd or 3rd AV blocks in foals (4, 5). At this specific dose, the effect on β1 and β2 receptors predominate and lead to increase in heart rate, cardiac output and myocardial contraction (6). This increase might be responsible for improvement in conduction and elimination of AV blocks (4). Intravenous epinephrine has had the potential to convert ventricular asystole to “rapid sinus rhythm” (7) and should be considered as emergency drug whenever atropine fails to convert advanced AV block into sinus rhythm. Epinephrine is derived from dopamine and both molecules are linked in their mechanism of action. At low doses (0.01 mg/kg), epinephrine principally acts on β1 and β2 receptors (6) and the same mechanism of action as dopamine might explain its success in the treatment of advanced AV blocks. According to their findings, the authors speculate that epinephrine and its biosynthetic precursor pathway molecules (dopamine, norepinephrine) might successfully treat advanced AV blocks when IV administrations of atropine is ineffective (7). Norepinephrine has been suggested as a suitable drug during cardiac arrest or CPR in clinical cases and experimental models (8, 9). Atropine as first treatment for advanced AV blocks needs to be considered; but whenever the drug fail to convert advanced AV block into sinus rhythm, administration of norepinephrine or its derivatives should be considered promptly.

The rhythm observed in this colt may be better defined as high grade 2nd degree AV block as ventricular activity was triggered after P waves and the QRS complexes remained of normal morphology. However, 3rd degree AV block remains a valid differential diagnosis for the observed rhythm. A definitive classification of the dysrhythmia could have been obtained with continuous ECG recording including several leads derivation. Unfortunately, valid record of the observed rhythm is not available, precluding a definitive classification. We are unaware of a duration of second degree AV block or the number of non-conducted P waves being part of the distinction between 2nd and 3rd degree AV block.

Advanced heart blocks during general anaesthesia of foals with or without uroperitoneum have been reported (4, 7, 10). Electrolyte imbalance such as hyperkalemia, hyponatremia and hypochloremia usually associated with ruptured urinary bladder do appear to precipitate cardiac arrhythmia. Much has been written about importance of hyperkalemia in the foal with uroperitoneum (11–13). The rise of extracellular plasma level reduces the resting potential across the cell membrane. An initial increase in tissue excitability is usually observed with mild hyperkalemia and followed by decreased excitability as plasma potassium level rises further. This leads to a reduction in conduction velocity at the sinus node, intraatrial and AV node. An additional increase in parasympathetic tone can further reduce conduction of the AV node and might induce complete AV blocks (14). Purkinje fibers seem to be even more sensitive to hyperkalemia than AV or sinus node, therefore precipitating the occurrence of AV blocks (15). Hyperkalemia should be treated promptly whenever diagnosed and fluid therapy should be initiated. The choice of NaCl 0.9% has been driven by the lower sodium and chloride venous content of the foal. Fluid therapy can be supplemented with glucose 2.5–5% to promote insulin production and consequent intracellular uptake of potassium ions. Insulin itself can be directly administered. Other treatment options include the administration of sodium bicarbonate administration (1–2 mEq/kg slow IV over 15 min). Bicarbonate stimulates the extracellular release of hydrogen ions and stimulates the intracellular uptake of potassium ions. This exchange maintains electroneutrality and consequently lowers serum potassium level (16). Calcium-gluconate (4 mg/kg slow IV over 10–20 min) can also be administered (17). It does not lower potassium plasma concentrations but calcium offers an indirect cardioprotective effect by increasing threshold voltage, restoring the normal resting membrane potential previously increased by hyperkalemia. The use of β2-adrenergic agonist drugs such as nebulised salbutamol or injected terbutaline have also been used successfully to decrease hyperkalemia (18, 19). The mechanism of action is thought to be linked to the stimulation of the Na^+^/K^+^ -ATPase. This case illustrates the difficulty in normalizing potassium plasma concentrations despite implementation of the above-mentioned treatments. In this case, it would have been wise to prolong the pre-operatory efforts of electrolyte stabilization and abdominal drainage. It is important to consider that the absence of hyperkalemia in foals with uroperitoneum does not exclude the occurrence of anaesthetic complications in the form of AV block or conduction block (13).

Sudden decrease of intraabdominal pressure when urine is drained rapidly has the potential to induce cardiovascular collapse. A vasovagal reflex may have been triggered by the release of urine by free flow from the abdomen. Decreased venous return to the heart may have been generated by massive inspiratory pressure through vena cava compression and sudden venous blood pooling in the mesenteric vasculature. This might have been the main trigger for advanced atrioventricular block in this case. Similar cardiovascular consequences (3rd degree AV block) have been reported after hot peritoneal lavage (20); the authors suggested that the peritoneal lavage had triggered a vasovagal reflex. It has been suggested to drain urine slowly to avoid atrioventricular block and arrhythmias (7, 11) and this would likely have been beneficial in the case reported here. The potential causes include dorsal recumbency, heat applied to the foal to prevent hypothermia, pulling on abdominal organs or intraabdominal pressure changes.

Atropine is the treatment of choice for many bradyarrhythmias such as vagally mediated bradycardia or bradyasystolic cardiac arrest (21). Atropine has been successful to treat 3rd degree AV block (20) but does not always seem effective to treat advanced AV block as illustrated in the present case. Paradoxical bradycardia or hypothermia are potential causes for the lack of efficacy of atropine. Those reasons do not to apply to this specific case and the explanation for treatment failure of atropine in patients encountering advanced AV-blocks remains unknown.

Another proarrhythmic factor that could have contributed to AV block in the present case was hypoxemia (22). To maintain an adequate level of saturated haemoglobin with oxygen, recruitment of collapsed alveoli by applying higher PIP together with PEEP and increasing the inspired fraction of oxygen was attempted. Unfortunately, those manoeuvres are likely detrimental to coronary perfusion which is essential to maintain adequate oxygen delivery to the heart and prevent myocardial hypoxia. Those manoeuvres have minor benefits as long as a high volume of intraabdominal fluids are exerting pressure on the diaphragm consequently compressing the lungs. An effective therapy will be the release of intraabdominal pressure. The anaesthetist and surgeon should coordinate this step and balance the slow release of intraabdominal fluids to enable lung expansion and prevent cardiovascular consequences related to the rapid release of intraabdominal pressure.

The analgesic protocol may have been suboptimal. The prolonged period of intraabdominal pressure could have contributed to increased sympathetic activity. When intraabdominal pressure due to uroperitoneum was released, the sympathetic stimuli has suddenly been released and might have triggered a reduction of nociception which potentiated the arrhythmogenesis.

The pathophysiology of cardiovascular complications during anaesthesia of the foal with uroperitoneum is likely to be multifactorial. The potential causes mentioned and the intrinsically immature sympathetic nervous system of neonates might induce detrimental cardiovascular consequences and promote life threatening cardiac arrhythmias.

After recovery, the discussion of whether the observed cardiac dysrhythmia could be called “cardiac arrest” was raised. According to the American Heart Association scientific statement, cardiac arrest is defined as “the cessation of cardiac mechanical activity, as confirmed by the absence of signs of circulation” (23). This suggests that the electrical activity without the presence of a pulse pressure could be called “cardiac arrest”. No arterial pressure waves were detected during prolonged advanced AV block and ETCO_2_ progressively decreased. Team training and preparation, uninterrupted chest compressions (“push hard, push fast”) at a rate of 100–120/minutes, ventilation provided by short breaths at a rate of 10–20/min and the administration of low-dose epinephrine (0.01 mg/kg IV or 0.1 mg/kg intratracheal) are considered key points for successful resuscitation during cardiac arrest. Those guidelines are the actual recommendations regarding CPR in the neonatal foal (24). Providing advanced life support through CPR is essential to rapidly restore return of spontaneous in case of cardiac arrest. Considering that cardiac mechanical activity was absent in the present foal and that CPR enabled restoration of normal sinus rhythm and adequate cardiovascular functions, it seems reasonable to call the observed clinical scenario “cardiac arrest”, consequence of advanced AV block. Advanced second AV blocks might be “the first step” to cardiac arrest because they might have dramatic cardiovascular consequences and should be considered and treated as cardiac arrest. It is interesting to note that Richardson and Kohn (10) observed severe cardiac arrhythmias in 9 foals undergoing halothane anaesthesia for uroperitoneum repair. They described 6 of these foals as having “3rd degree AV or cardiac arrest” while the authors would suggest advanced 3rd degree AV block is a cardiac arrest.

Another terminology discussion was pertaining to the term asystole. “Asystole” (from Greek: “a” =privative prefix, “systole” =contraction) has been defined as “the complete lack of electrical activity in the heart” (25) and is colloquially called “flat-line”. Jacobs et al. (23) stated that “although a specific definition of asystole is desirable, no consensus agreement was reached on either a specific duration (e.g., 30 s) or heart rate (e.g., <5 bpm) to define asystole versus bradycardia/pulseless electrical activity”. Etymologically speaking asystole refers to lack of mechanical activity. Lack of electrical activity always leads to lack of mechanical activity but the reverse is not always true. These definitions are centred on electrical or mechanical ventricular activity and AV blocks are difficult to fit here *sensu stricto* due to the presence of electrical and likely mechanical atrial activity in these cases.

## Conclusion

Based on this clinical scenario, we do believe that advanced AV block is a severe cardiovascular dysrhythmia that, according to the revised definitions, could be called and treated as cardiac arrest. Foals with uroperitoneum can encounter severe disturbances in cardiovascular homeostasis through advanced AV block which might be life threatening. Consequently, the anaesthesiologist should work with the internist and surgeon in the preoperative and operative management and be ready to provide advanced life support any time severe arrhythmias occur. Treatment toward the cause of the blocks should be favoured. This will prevent cardiac arrest or asystole and decrease morbidity and mortality.

## Ethics Statement

Signed owner consent was obtained for publication.

## Author Contributions

VM was responsible for the anaesthetic management of the foal. He wrote the manuscript and revised the definitions presented in the paper. AM took part to the anesthetic management of the foal. He recorded all datas and participated to the redaction of the manuscript. NF was responsible for the medical management of the case and for the pre-and post-operative care. She participated the redaction of the manuscript. CN was responsible for the medical management of the case. He participated to the redaction of the manuscript and revised the definitions presented in the paper.

## Conflict of Interest Statement

The authors declare that the research was conducted in the absence of any commercial or financial relationships that could be construed as a potential conflict of interest.

The reviewers TM, AB-S and handling Editor declared their shared affiliation.
